# Supplementary material for: Parylene photonics: a flexible, broadband optical waveguide platform with integrated micromirrors for biointerfaces
Source: Microsyst Nanoeng. 2020 Sep 21;6:85. doi: 10.1038/s41378-020-00186-2 (PMC8433189; doi:10.1038/s41378-020-00186-2)
Supplement: Supplementary file 1 — Supplementary Information [file 41378_2020_186_MOESM1_ESM.docx]

**Supplementary Material**

**Parylene Photonics: A Flexible, Broadband Optical Waveguide**  **Platform with Integrated Micromirrors for**  **Biointerfaces**

Jay W. Reddy, Maya Lassiter, Maysamreza Chamanzar*

*Department of Electrical and Computer Engineering, Carnegie Mellon University, Pittsburgh, USA*

**Appendix I: Crosstalk Simulation**

Parylene C and PDMS are excellent candidate materials for a biophotonic platform due to their biocompatibility, flexibility, transparency in the visible range, and ease of processing using traditional microfabrication techniques. Additionally, Parylene C has a high refractive index among polymer materials (n = 1.639), whereas PDMS has a low refractive index (n = 1.4), allowing for devices with high index contrast. Compared to dielectric materials such as silicon nitride (n = 2.05 at λ = 450 nm)^66^, however, Parylene C provides much less index contrast. A simulation is performed to show that the index contrast in Parylene C/PDMS is sufficient for a high-density photonic platform. A concern of densely integrated waveguide arrays is the crosstalk between adjacent waveguide channels due to evanescent field coupling between the channels. As waveguides become smaller, the optical modes become less confined, and more optical power propagates in the cladding. As a result, this optical power can be coupled into adjacent waveguide channels.

The simulation is performed using a bidirectional eigenmode expansion (EME) solver (*Lumerical MODE Solutions 2018b, Lumerical Inc., USA)*. The simulated waveguide has a cross-section of 1 µm x 1 µm (Fig. S1a). Two parallel waveguides with 1 µm spacing are simulated over a propagation length of 5 cm (Fig. S1b). The input optical power in the “input waveguide” is normalized to 1, and the proportion of optical power in the adjacent “probe waveguide” is measured. Over the 5 cm propagation length, increasing power is observed in the probe waveguide due to evanescent field coupling (Fig. S1c,d). Over a 5 cm length, a total crosstalk of -32.86 dB is observed.


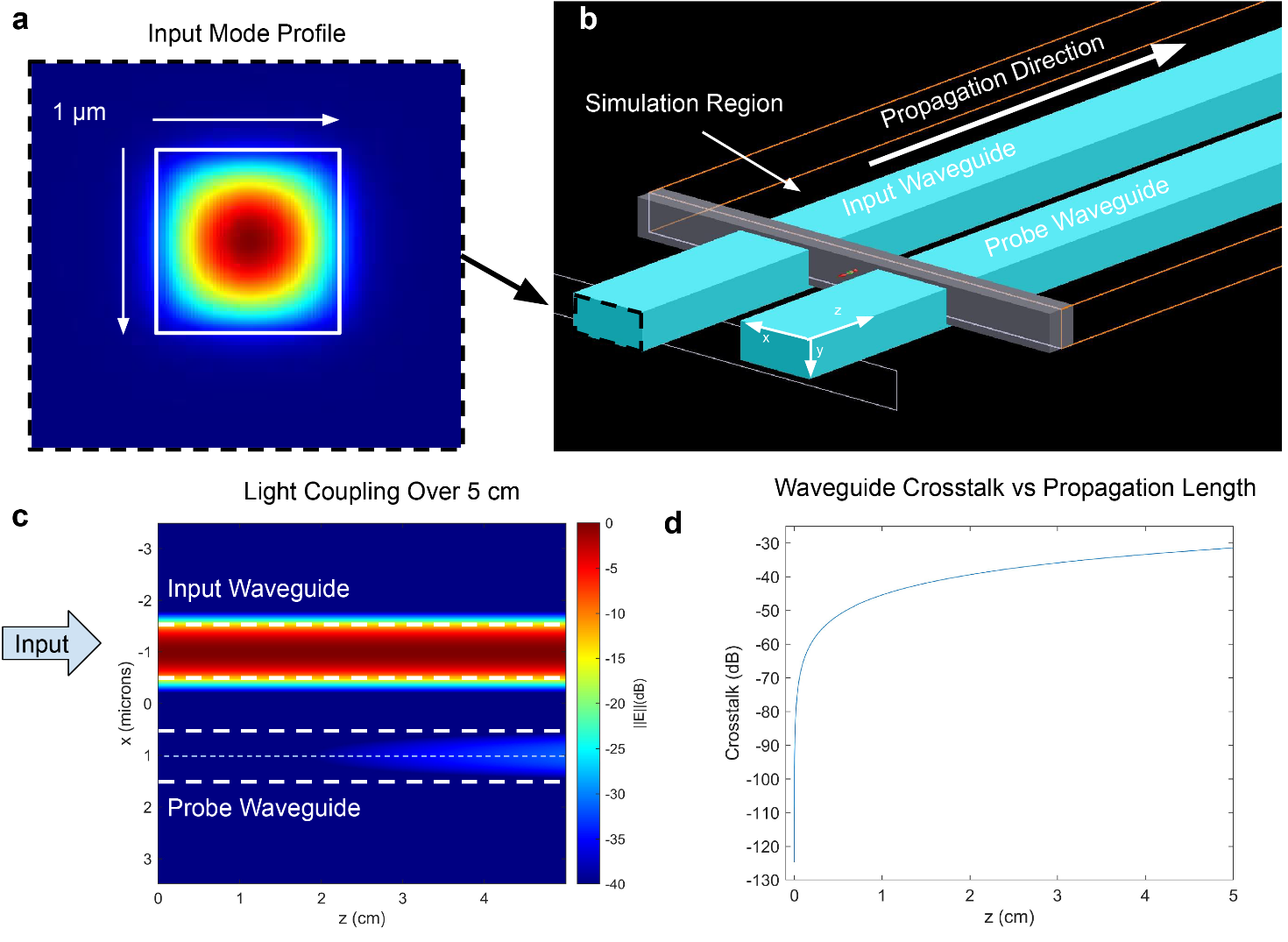


**Figure S1:** Miniaturized high-density Parylene photonic waveguide arrays with low crosstalk. a) The optical mode profile of a 1 µm x 1 µm waveguide showing the fundamental mode at λ = 450 nm. b) Schematic of two parallel waveguides for crosstalk analysis. The input waveguide (left) is powered while the probe waveguide (right) is monitored over a propagation length of 5 cm. c) Electric field profile of the parallel waveguide structure over a 5 cm propagation distance. Light coupling into the probe waveguide is observed. d) Plot of crosstalk in the probe waveguide vs propagation length in decibels (dB). At 5 cm, a crosstalk of -32.86 dB is observed.
